# Orf disease in a patient with rheumatoid arthritis

**DOI:** 10.31138/mjr.29.2.89

**Published:** 2018-06-29

**Authors:** Markos Kostopoulos, Charalampos Gerodimos, Eirini Batsila, Christina Kalinou, Panagiotis Athanassiou

**Affiliations:** 1 Rheumatology Department, General Hospital of Thessaloniki “Agios Pavlos”, Thessaloniki, Greece,; 2 Consultant General Practitioner, Thessaloniki, Greece,; 3 Consultant Dermatologist, Thessaloniki, Greece

**Keywords:** Orf disease, rheumatoid arthritis, immunosuppression

## Abstract

Orf disease is a viral infection, affecting patients being involved in the care of livestock either professionally or habitually. It is also known as ecthyma contagious, contagious pustular dermatitis or infectious euphoria being a rare zoonotic disease caused by an epitheliotropic DNA virus from the parapoxvirus group. We report a case of Orf disease affecting the hand of a patient with rheumatoid arthritis (RA) on treatment with methotrexate and adalimumab, an anti-tumor necrosis factor biological agent. The patient was successfully treated with doxycycline, while immunosuppressive treatment was discontinued.

## INTRODUCTION

Orf disease is a viral disease affecting mainly farmers being involved in the care of livestock. The disease is a zoonotic disease being transmitted from animal to animal by contact. It affects people who are in contact with animals, either professionally or habitually; the case of sheep being mostly reported in the literature. It is a self-remitting disease.

Nowadays, rheumatoid arthritis (RA) is treated by immunosuppressive agents, particularly anti-TNF biologics. Patients may become immunosuppressed and they may develop infections. The case of a farmer with RA on treatment with immunosuppressants, who developed Orf disease which was successfully treated, is described.

## CASE REPORT

**Figures 1–4:**
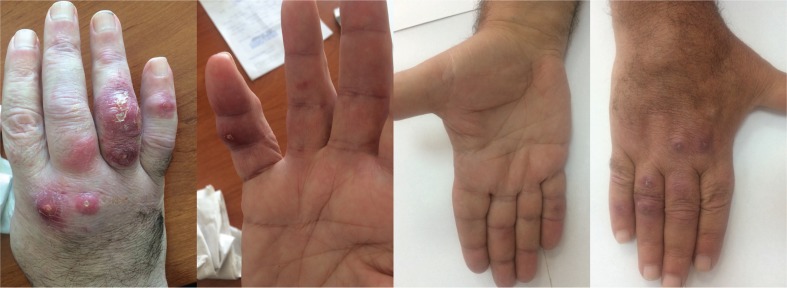
Orf disease. Nodular lesions of the dorsal (1) and palmar (2) surface of the right hand two months after the eruption. Healed lesions with scarred tissue two months after the discontinuation of immunosuppressive drugs (3,4).

A 65-year old man presented to the outpatient clinic complaining of severe pain in his right hand, which developed over the course of two months and progressively deteriorated. The patient had a 3-year history of anti-CCP positive and RF positive RA. The clinical examination revealed a swollen and warm hand with tender and erythematous nodules. These lesions exhibited spontaneous outflow of serous fluid. The lesions evolved after exposure to livestock. The patient was a farmer and had a farm in which he looked after sheep. The first lesion started as a blister on the palmar surface of the fifth finger of the right hand, with new lesions gradually expanding to the dorsal surface (**Figure 1**). Body temperature and vital signs were normal. Physical examination revealed absence of tender and swollen joints, with RA being in remission.

The patient was examined by a dermatologist, and chilblains were diagnosed. Local medication was initiated, and amoxicillin with clavulanic acid was administered for a period of 20 days. However, the lesion did not improve. The immunosuppressive treatment for RA consisted of methotrexate 12.5mg orally weekly, methylprednisolone 4mg daily and adalimumab 40mg subcutaneously every other week. Other medications included amiodarone and dicumarol for atrial fibrillation, lorazepam and enalapril for arterial hypertension, and calcium plus vitamin D, as the patient was on long-term treatment with corticosteroids. Initial laboratory testing showed mild leukocytosis (WBC 11,000 × 10^9^/L) with elevated markers of inflammation (ESR 42mm/h and CRP 1.9mg/dL). Serological testing for Brucella species, hepatitis viruses and tuberculin skin test were all negative. Ziehl-Neelsen stain and cultures of the fluid for common bacteria, fungi and mycobacteria were negative.

A new dermatological assessment was made, and the lesions were described as target-like papillary nodules with exudative fluid and erythematous margins.

The diagnosis of Orf disease in an immunocompromised patient was made in a patient with a history of occupational exposure to sheep. Subsequently, all immunosuppressive drugs were discontinued except methylprednisolone, which was increased from 4 to 6mg in order to keep RA in remission. In parallel, surgical debridement of the inflamed cutaneous area was performed. Ten days after the aforementioned interventions, the patient’s hand improved. Concurrently inflammatory markers improved, ESR and CRP being 31 mm/h and 0.5 mg/dL, respectively. Two months later, the nodules improved, leaving a scar at the initial sites of inflammation (**Figure 2**). RA remained in remission, and methotrexate was reintroduced at the initial dose, while methylprednisolone was tapered to 4mg daily.

## DISCUSSION

Orf disease, also known as ecthyma contagious, contagious pustular dermatitis or infectious euphoria, is a rare zoonotic disease caused by an epitheliotropic DNA virus from the parapoxvirus group.^1^ Carrier animals (goats and sheep) transmit the virus to humans through direct contact, especially during milking from infected breasts.^2^ Transmission also occurs indirectly from the environment after inoculation of infected materials on plants.^2^ Areas of skin or mucosal discontinuation are entrance gates where the virus is locally multiplied, creating the characteristic lesions of the disease.^1^ They tend to appear on the hands and the fingers, and, rarely, on the face, nostrils, tongue, eyelids and perianally.^1^

The first phase of the disease is characterized by the appearance of a small papule one week after exposure to the virus. In the second phase, the lesions enlarge and take on an iris-like shape with a central red nodule, a surrounding white circle and an erythematous exterior margin. In the third phase, the lesions enlarge rapidly and have an exudative appearance. The fourth phase is regenerative, when the lesion takes on black spots and exhibits a thin crust. The fifth phase is papillomatous, with very small papillomas that can be seen on the lesion. The last phase is regression.^1^

Contact with sheep or goats, a history of work in the slaughtering industry, clinical appearance and epidemiologic data are important for diagnosis which is made clinically, based on history of contact with infected animals or environment, accompanied by the typical nodular target-like lesions. The diagnosis is confirmed only in severe cases with histopathologic studies, electron microscopy and virus isolation by PCR.^3^ Differential diagnosis includes herpetic whitlow, cowpox, milker’s nodule, cat-scratch disease, anthrax, tularemia, deep fungal infections, tuberculosis, atypical mycobacterial infections, syphilitic chancre, sporotrichosis, keratoacanthoma, pyogenic granulomas and malignant tumors.^4^ In our patient, there was no need for further examinations, as the clinical findings and the history of exposure were typical. The disease usually follows a benign course and is self-limited within 4–8 weeks, making medical and surgical interventions unnecessary.^5^ In immunocompromised patients, the disease may be persistent, requiring medical treatment such as antiviral therapy (cidofovir), corticosteroid use, cryotherapy and surgical resection because of the risk of serious secondary infections.^5^–^8^

In the case described herein, the patient suffered from RA and was treated with immunosuppressive drugs, namely methotrexate, adalimumab and corticosteroids. This treatment approach led to remission of RA, but was the cause for a potentially self-limiting disease to become chronic. For this reason, patients should be monitored regularly and, in case of infection, the administration of synthetic and biological DMARDs should be modified or discontinued, the infection being treated simultaneously.^9^ In the case of our patient, discontinuation of immunosuppressive drugs was enough for the cure of Orf disease.

## CONCLUSION

Detailed history and clinical examination are vital for the diagnosis and treatment of atypical or uncommon infections such as Orf disease in immunosuppressed patients. Orf disease is benign and self-limiting, but the administration of immunosuppressive therapy can lead to chronicity with the risk of secondary bacterial infection. Awareness of the disease and a high index of suspicion is important in order to avoid inappropriate treatment.

## ABBREVIATIONS

RA: Rheumatoid arthritis
CRP: C-reactive protein
ESR: Erythrocyte sedimentation rate
WBC: White blood cells
PCR: Polymerase chain reaction
DMARDs: Disease Modifying Anti Rheumatic Drugs
